# Spatiotemporal Variations in Co-Occurrence Patterns of Planktonic Prokaryotic Microorganisms along the Yangtze River

**DOI:** 10.3390/microorganisms12071282

**Published:** 2024-06-24

**Authors:** Wenran Du, Jiacheng Li, Guohua Zhang, Ke Yu, Shufeng Liu

**Affiliations:** 1 School of Environment and Energy, Peking University Shenzhen Graduate School, Shenzhen 518055, China; 2 The Key Laboratory of Water and Sediment Sciences, Ministry of Education, Department of Environmental Engineering, Peking University, Beijing 100871, China; 3 College of Resources and Environmental Sciences, China Agricultural University, Beijing 100193, China

**Keywords:** bacterial community, archaeal community, non-random co-existence networks, biogeography, landforms, seasonal variation, the Yangtze River

## Abstract

Bacteria and archaea are foundational life forms on Earth and play crucial roles in the development of our planet’s biological hierarchy. Their interactions influence various aspects of life, including eukaryotic cell biology, molecular biology, and ecological dynamics. However, the coexistence network patterns of these microorganisms within natural river ecosystems, vital for nutrient cycling and environmental health, are not well understood. To address this knowledge gap, we systematically explored the non-random coexistence patterns of planktonic bacteria and archaea in the 6000-km stretch of the Yangtze River by using high-throughput sequencing technology. By analyzing the O/R ratio, representing the divergence between observed (O%) and random (R%) co-existence incidences, and the module composition, we found a preference of both bacteria and archaea for intradomain associations over interdomain associations. Seasons notably influenced the co-existence of bacteria and archaea, and archaea played a more crucial role in spring as evidenced by their predominant presence of interphyla co-existence and more species as keystone ones. The autumn network was characterized by a higher node or edge number, greater graph density, node degree, degree centralization, and nearest neighbor degree, indicating a more complex and interconnected structure. Landforms markedly affected microbial associations, with more complex networks and more core species found in plain and non-source areas. Distance-decay analysis suggested the importance of geographical distance in shaping bacteria and archaea co-existence patterns (more pronounced in spring). Natural, nutrient, and metal factors, including water temperature, NH_4_^+^-N, Fe, Al, and Ni were identified as crucial determinants shaping the co-occurrence patterns. Overall, these findings revealed the dynamics of prokaryotic taxa coexistence patterns in response to varying environmental conditions and further contributed to a broader understanding of microbial ecology in freshwater biogeochemical cycling.

## 1. Introduction

Bacteria and archaea, distinguished as some of the earliest life forms on our planet, play foundational roles in Earth’s biological hierarchy. These two groups of microorganisms exhibit distinct morphological, physiological, and evolutionary characteristics. Bacteria play diverse roles in natural environments, such as participating in the decomposition and cycling of organic matter and maintaining ecological balance, which are particularly vital to biogeochemical fluxes and nutrient cycling in river ecosystems [1,2]. Archaea, considered the third domain of life separate from bacteria and eukaryotes, are essential regulators of global carbon and nitrogen cycles [3,4]. Previous studies have advanced our understanding of the prokaryotic microbial distribution and contributing factors affecting bacterial and archaeal communities across a variety of ecosystems, including natural rivers [5], estuaries [6], and urban rivers [7]. Comprehensive studies on the microbiome along the Yangtze River, the world’s third-largest river, have unveiled the spatial and temporal dynamics of the bacterial and archaeal communities in this huge fluvial system [8,9].

The mechanisms underpinning species co-existence are elucidated via network-based analyses regarding non-randomness, keystone species, modularity, and topological characteristics [10,11]. The interactions between bacteria and archaea are of particular importance and significantly influence various aspects of eukaryotic cell biology, molecular biology, ecological dynamics, and evolutionary processes, mediating microbial functions [12,13]. However, coexistence patterns between bacteria and archaea in river ecosystems have received limited attention, with most studies focusing on soils or estuarine sediments. For instance, a study on network interactions between bacteria and archaea in the alpine grassland of the Tibetan Plateau highlighted the significant impact of soil pH on the topological characteristics and stability of microbial co-occurrence networks, with archaea rather than bacteria playing a more central role in the network [14]. Another recent study in estuarine sediments has revealed that higher salinity favored intra-domain (archaea–archaea and bacteria–bacteria) over interdomain interactions, suggesting a critical role of intra-domain relations in shaping microbial community structure [15]. The interactions and coexistence patterns between bacteria and archaea may vary across different ecosystems. River ecosystems, bridging continental and marine biogeochemical cycles, are crucial for water circulation, material transport, civilization emergence, and socio-economic development [16,17]. Riverine bacteria and archaea are critical in nutrient cycling, energy transfer, and pollutant decomposition [18], and the importance of investigating their coexistence patterns in riverine habitats should be emphasized. However, the complexities of bacterial and archaeal co-existence remain inadequately explored in a large river system extending from source to estuary, which poses challenges to effective river management.

In this study, we conducted a synchronous sampling over a 6000 km reach of the Yangtze River, Eurasia’s largest river flowing through diverse terrains with significant environmental gradients. By collecting approximately 100 water samples in two seasons for 16S ribosomal RNA (rRNA) gene amplicon sequencing and physicochemical analysis, we aimed to: (a) elucidate the non-randomness of bacterial and archaeal co-existence patterns within this large river; (b) assess seasonal and geographic variations of network topology; and (c) identify the key environmental factors influencing the co-existence patterns. Based on a large-scale survey across the river’s continuum, this study outlined the spatiotemporal dynamics and environmental drivers of bacteria and archaea co-existence patterns and offered essential insights into freshwater microbial ecology for decision-makers.

## 2. Materials and Methods

### 2.1. Study Area and Sample Collection

**Figure 1:**
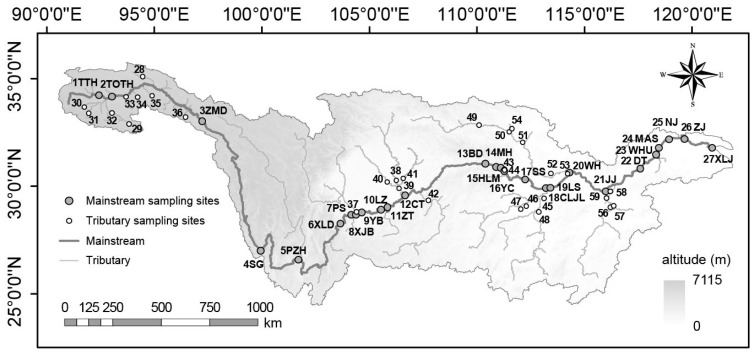
Study area and the sampling sites. Detailed information is summarized in Table S1.

The Yangtze River, the world’s third-longest, originates from the Tangula Mountain on the Qinghai-Tibet Plateau and flows towards the East China Sea. In this study, we collected water samples from national hydrologic stations along a 6000 km continuum of the Yangtze River (Figure 1). The first-stage sampling was conducted in March (spring) and October (autumn) of 2014 and the second-stage sampling was performed in July 2017, which included 27 sites in the mainstream and 32 sites in some major tributaries. Detailed sampling procedures have been reported by Liu et al. [8]. Moreover, 12 sites are located in the source area (nos. 1–3 and 28–36), and 47 sites are located in the non-source area (nos. 4–27 and 37–59) of the Yangtze River. Considering terrain variations, those sites in the Yangtze River mainstream can be further categorized into the plateau (nos. 1–3), mountain/hill (nos. 4–8 and 13–17), basin (nos. 9–12), and plain (nos. 17–27) groups [8].

### 2.2. Physicochemical Analysis

Geographic coordinates for each sampling site were recorded using a GPS device. A geographic (geodetic) distance matrix among all the sites was calculated using the “*geoXY*” function of the SoDA package in R language. Environmental factors consisted of a series of physiochemical parameters that were measured according to the standard methods and the elevation that was recorded by GPS. Water temperature (WT) and pH were in-situ measured using a multi-parameter online analyzer. Chemical oxygen demand (COD_Mn_), ammonium nitrogen (NH_4_^+^-N), nitrate nitrogen (NO_3_^−^-N), total nitrogen (TN), and total phosphorus (TP) were, respectively, determined based on GB 11901-89, GB 11892-89, HJ 535-2009, HJ/T 346-2007, HJ 636-2012, and GB 11893-89 issued by Ministry of Ecology and Environment of China (https://www.mee.gov.cn/ accessed on 21 April 2024). Concentrations of some metals including calcium (Ca), iron (Fe), aluminum (Al), nickel (Ni), and cadmium (Cd) were measured using ICP-MS or ICP-OES according to the methods of our previous study [19].

### 2.3. DNA Extraction and Illumina Sequencing

For each sample, total genomic DNA was extracted from liquid nitrogen-shattered membranes using FastDNA^®^SPIN Kit for Soil. DNA quality and quantity were evaluated using a NanoDrop ND-2000 spectrophotometer. Duplicates of each sample were processed to ensure enough high-quality DNA. Archaeal 16S rRNA genes were PCR-amplified using the barcoded primer pairs 524F-10-ext (5′-TGYCAGCCGCCGCGGTAA-3′) and Arch958R-mod (5′-YCCGGCGTTGAVTCCAATT-3′) [20]. PCR mixtures (20 µL) were set up in triplicate, containing 10 ng of template DNA, 4 μL of 5× FastPfu Buffer, 2 μL of 2.5 mM dNTPs, 0.4 μL of bovine serum albumin, and 0.8 μL of each primer (5 μM). PCR reactions contained an initial denaturation at 95 °C for 3 min, 37 cycles of denaturation at 95 °C for 30 s, annealing at 55 °C for 30 s, elongation at 72 °C for 45 s, and a final extension at 72 °C for 10 min. For bacteria, the V4–V5 region of bacterial 16S rRNA gene was PCR-amplified using the barcoded primer pairs 515F (5′-GTGCCAGCMGCCGCGG-3′) and 907R (5′-CCGTCAATTCMTTTRAGTTT-3′) [21]. PCR reactions were performed in triplicate with a 20 μL mixture containing 10 ng of template DNA, 4 μL of 5× FastPfu Buffer, 2 μL of 2.5 mM dNTPs, 0.4 μL of FastPfu polymerase, and 0.8 μL of each primer (5 μM). The amplification process contained an initial denaturation at 95 °C for 2 min, 25 cycles of denaturation at 95 °C for 30 s, annealing at 55 °C for 30 s, extension at 72 °C for 30 s, and a final extension at 72 °C for 5 min.

Amplicon purification was done using the AxyPrep DNA Gel Extraction Kit. Purified amplicons were pooled in equimolar amounts and sequenced with a paired-end strategy (2 × 250 bp for bacteria and 2 × 300 bp for archaea) on the Illumina MiSeq platform (Majorbio Company, Shanghai, China). All the raw sequences have been provided by previous studies and deposited on the NCBI website [8,9].

### 2.4. Bioinformatic Analysis

The paired-end reads of bacterial or archaeal 16S rRNA genes were merged and processed to delete the low-quality reads according to previous studies [8,9]. Operational taxonomic units (OTUs) were generated by UPARSE using a sequence similarity cutoff of 97% [22]. Chimeras were then removed by UCHIME [23]. The taxonomic information of each 16S rRNA gene sequence was obtained by RDP Classifier [24] by searching against the Greengenes database [25] for bacteria and the SILVA small subunit database [26] for archaea.

### 2.5. Statistical Analysis

Co-existence networks of bacterial and archaeal OTUs were constructed separately for spring, autumn, and the two seasons using Hmisc, igraph, and vegan packages in R [27]. To lower the bias, Spearman’s correlation coefficients were calculated based on the relative abundance between OTUs that appeared in at least 30% of the samples. The robust (Spearman’s *r* > 0.6) and significant (Benjamini–Hochberg corrected *p* < 0.01) correlations were then visualized and analyzed for network modularity using Gephi v0.9.1 (https://gephi.org/, accessed on 21 April 2024). Besides, 1000 Erdös–Rényi random networks with the same number of nodes and edges as the realistic networks were generated for comparative analysis [28]. Moreover, a total of 12 typical topological features were calculated for the subnetwork by following the procedures of two previous studies [10,29]. Group differences were evaluated using Wilcoxon rank-sum tests, and Spearman’s correlations were used to explore the relations between environmental factors and network topological features. Moreover, statistics for comparisons of the observed (O) and random (R) co-existence incidences of taxa were done as previously suggested [28]. The O/R ratio, representing the divergence between observed (O%) and random (R%) co-existence incidences, quantified the non-randomness of interactions. A ratio > 1 signified the observed co-existence rates exceeding random chance.

One-way analysis of variance (one-way ANOVA) and Wilcoxon rank-sum tests were utilized to assess the significance of differences among groups using the stats package in R. The distance-based redundancy analysis (db-RDA) [30] was conducted to elucidate the impact of environmental factors on bacterial and archaeal OTUs that occurred in the co-occurrence network based on the Bray–Curtis dissimilarities by using the ‘*capscale*’ function in the R vegan package [31]. The distance-decay patterns were analyzed by Mantel tests to examine the correlations between geographic distances and network properties (using Bray–Curtis distance matrices) with 999 permutations in the vegan package in R.

## 3. Results

### 3.1. Bacteria–Archaea Co-Occurrence Network Structure

**Figure 2:**
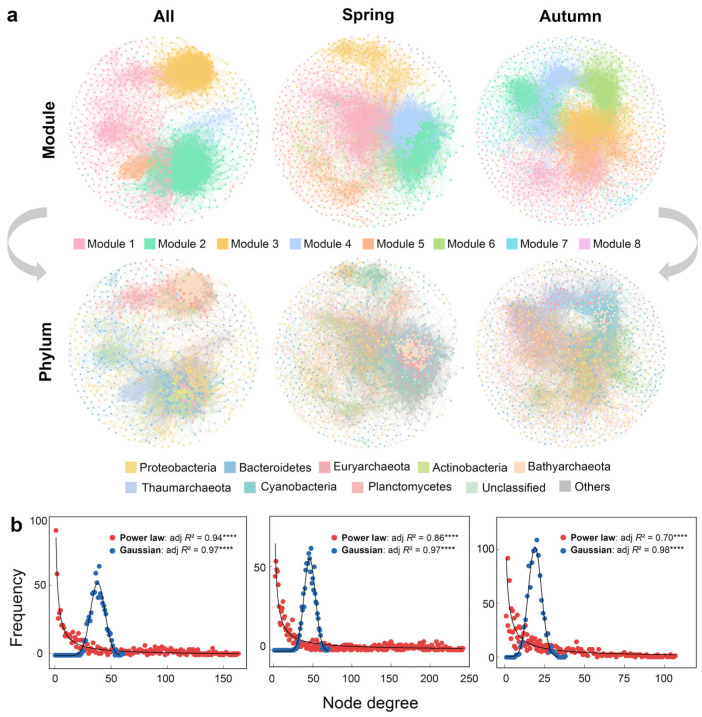
Metacommunity co-existence of planktonic prokaryotic taxa in the Yangtze River. (**a**) Bacterial and archaeal networks were constructed based on Spearman’s rank correlations between all the available OTUs in spring, autumn, or both seasons. Nodes are colored by modularity (upper panels) and phylum-level taxonomy (lower panels). Each connection stands for a strong (Spearman’s *r* > 0.6) and significant (Benjamini–Hochberg corrected *p* < 0.01) correlation. The size of each node is proportional to the degree. (**b**) Node degree distributions for the prokaryotic co-existence networks (in red) and the 1000 Erdös–Rényi random networks (in blue). Black solid lines denote the power law and Gaussian best fits (**** *p* < 0.0001) for the degree distribution from the realistic networks and the equally sized Erdös–Rényi random networks, respectively.

To analyze the co-occurrence patterns between bacterial and archaeal communities in the Yangtze River water, networks were constructed based on the strong (Spearman’s *r* > 0.6) and significant (false discovery rate adjusted *p*-value < 0.01) correlations between all available OTUs in spring, autumn and both seasons (Figure 2a). The network of both seasons consisted of 838 nodes and 16,309 edges, the spring network was comprised of 971 nodes and 22,394 edges, and the autumn network consisted of 1071 nodes and 10,192 edges. The small-world coefficients of planktonic prokaryotic co-existence networks in the Yangtze River were much greater than 1, indicating the significant modular structure of networks (more pronounced in autumn) [32] (Table 1). Compared to networks of other studies, we found that the small-world coefficients of the co-existence networks of planktonic prokaryotic communities in the Yangtze River were lower than those of the Jiulong River water, which may be due to the fact that Jiulong River water was affected by persistent organic pollutants for a long time, and some indicator bacteria such as Clostridiales were abundant in the river for the formation of more significant network modularity [28]. The small-world property of the planktonic co-existence networks in the Yangtze River is much higher than those of marine microbial networks and other biotype networks, but lower than those of the human social networks (Table 1). The node degree distribution of all the three networks of the Yangtze River obeyed a power law pattern (all: adj. *R*^2^ = 0.94, *p* < 0.0001; spring: adj. *R*^2^ = 0.86, *p* < 0.0001; autumn: adj. *R*^2^ = 0.70, *p* < 0.0001), while the Gaussian distribution (all: adj. *R*^2^ = 0.97, *p* < 0.0001; spring: adj. *R*^2^ = 0.97, *p* < 0.0001; autumn: adj. *R*^2^ = 0.98, *p* < 0.0001) was observed for the Erdös–Rényi random networks with an identical size (Figure 2b). This revealed that bacterial and archaeal OTUs formed scale-free network structures with non-random co-existence patterns [10,28].

Interestingly, we found that bacteria and archaea in the Yangtze River water tended to exhibit intra-domain interactions in the major module in both seasons. For example, in the spring network, more than 95.86% of the nodes in Module 1, Module 5, Module 7, and Module 8 were predominantly represented by OTUs of bacterial phyla like Proteobacteria, Bacteroidetes, and Actinobacteria. Similarly, in the autumn network, over 81.11% of the nodes in Module 1, Module 3, Module 5, and Module 7 were represented by OTUs of similar bacterial phyla. In a parallel manner, more than 83.88% of the members in Module 2, Module 3, Module 4, and Module 6 of the spring network and over 76.22% of the members in Module 2, Module 4, Module 6, and Module 8 in the autumn network were represented by OTUs of archaeal phyla such as Bathyarchaeota, Thaumarchaeota, and Euryarchaeota. This could be further confirmed by the O/R ratios, which were used to reflect the incidence of co-existence patterns between OTUs from the same and different phyla (Table 2). Similar to the module patterns in the networks, the O/R ratio analysis revealed a predominant tendency for both bacterial and archaeal OTUs that mostly co-existed with other OTUs in their own domains. These results were observed in the networks of spring, autumn, or both seasons. For bacterial communities in the overall network of both seasons, Actinobacteria OTUs tended to co-exist with seven other major bacterial phyla such as Proteobacteria, Bacteroidetes, Cyanobacteria, Planctomycetes, Chloroflexi, Verrucomicrobia, and Gemmatimonadetes more often than expected, as revealed by high O/R ratios (1.13–4.35). As for archaea, Bathyarchaeota OTUs showed the most extensive intra-phylum connections (O% = 10.07%, O/R-ratio = 15.50) and had the most inter-phylum linkages with Euryarchaeota (O% = 9.47%, O/R-ratio = 4.69), Thaumarchaeota (O% = 2.85%, O/R-ratio = 2.19), and YNPFFA (O% = 0.93%, O/R-ratio = 11.94). The network analysis also revealed significant seasonal variations of co-existence patterns. Compared with the autumn network in which bacterial and archaeal OTUs occupied 69.77% and 30.23% of the nodes, respectively, there was a great increase for archaeal OTUs that constituted 47.78% of the nodes in the spring network (Figure 2a), which suggested a more important role for archaea in spring rather than in autumn. The results for intra- and inter-phylum co-existence (Table 2) were also consistent with this finding.

### 3.2. Keystone Species and Their Taxonomic Distributions

**Figure 3:**
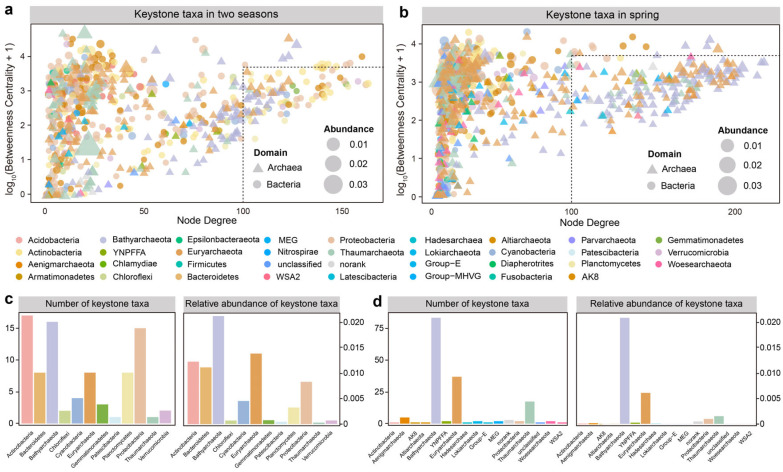
Identification of the keystone taxa in bacterial and archaeal networks for two seasons (**a**) and spring (**b**). Dot and triangle plots show the node degree and betweenness centrality of bacterial and archaeal OTUs in the networks, respectively. OTUs with high node degrees (>100) and low betweenness centrality values (<5000) were considered the keystone taxa [10]. The dot and triangle points are colored by the phylum-level taxonomy, and their areas are proportional to the mean relative abundances of OTUs. The colored bar charts exhibit the number and relative abundances of the keystone taxa in each phylum for two seasons (**c**) and spring (**d**). “MEG”: miscellaneous Euryarchaeotal group; Group-E: marine benthic group-E.

Keystone species are essential for the preservation of the microbial community structure and functionality and were identified based on the topological features of the nodes, i.e., high node degree (>100) and low betweenness centrality values (<5000) [10] (Figure 3). We identified 85 keystone species in the overall network of two seasons, in which bacterial species accounted for 54.32% of the total abundance, including members belonging to Actinobacteria (17 OTUs, the sum of relative abundances: 0.012), Bacteroidetes (8 OTUs, 0.011), and Proteobacteria (15 OTUs, 0.008). Archaeal keystone OTUs included members of phylum Bathyarchaeota (16 OTUs, 0.021) and Euryarchaeota (8 OTUs, 0.014). In the spring network, 164 out of the 167 keystone species were archaeal OTUs mainly belonging to Bathyarchaeota (86 OTUs, 0.201), Euryarchaeota (38 OTUs, 0.061), and Thaumarchaeota (18 OTUs, 0.016), which accounted for 95.93% of the total abundance and further suggested a crucial role for archaea within the spring network. With the same filter threshold applied for the autumn network, no keystone species were found.

### 3.3. The Topological Properties of the Co-Occurrence Networks

**Figure 4:**
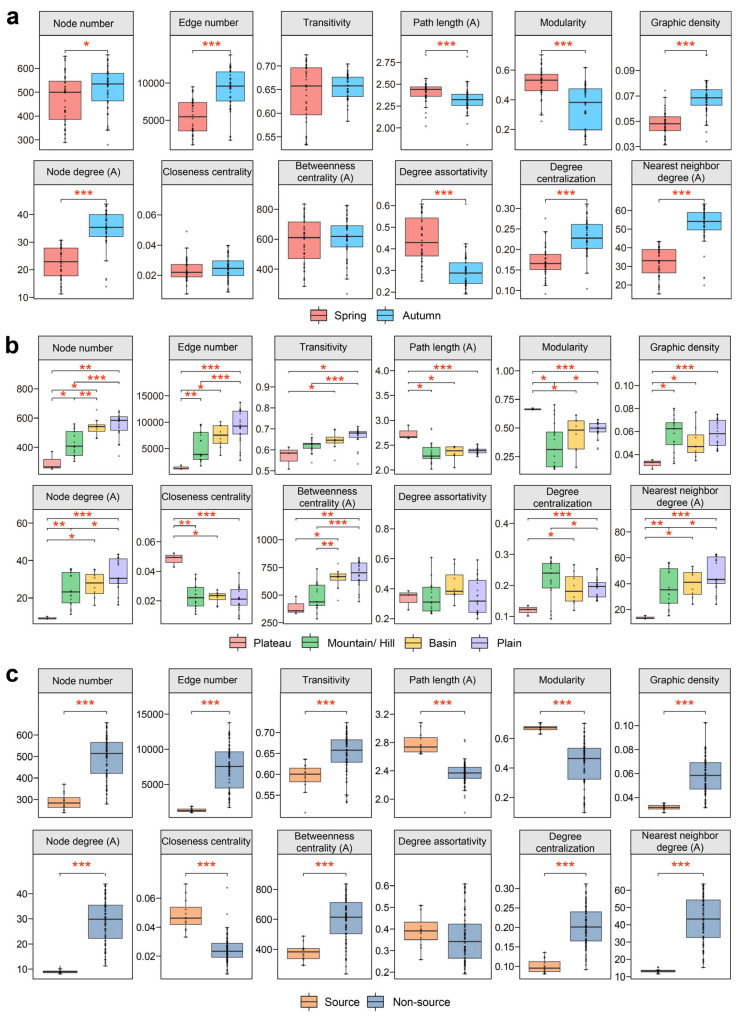
Boxplots displaying the comparisons of network topological features between spring and autumn (**a**), among plateau, mountain/hill, basin, and plain regions (**b**), and between the source and non-source regions (**c**). All the asterisks denote the significance of Wilcoxon rank-sum tests (*** *p* < 0.001, ** 0.001 < *p* < 0.01, and * 0.01 < *p* < 0.05). The “A” in the brackets means “average”.

To further analyze the properties of bacteria–archaea co-existence networks, the significance tests were conducted on the topological features across two seasons and different landform regions (Figure 4). The analysis contrasting the 12 network-level topological features uncovered significant seasonal and spatial variations in the network structure. Specifically, the autumn network displayed a higher number of nodes and edges, greater graph density, average node degree, degree centralization, and average nearest neighbor degree compared to the spring network (Wilcoxon rank-sum tests, *p* < 0.05), indicating a more complex and interconnected structure in autumn (Figure 4a). The spring network was characterized by a larger average path length, greater modularity, and a higher degree of assortativity (Wilcoxon rank-sum tests, *p* < 0.001). Furthermore, the network complexity and connectivity were significantly lower in plateau and source areas, as evidenced by lower numbers of nodes and edges, transitivity, graph density, average node degree, degree centralization, and average nearest neighbor degree (Wilcoxon rank-sum tests, *p* < 0.05). In contrast, the plain and non-source areas demonstrated notably higher values in these parameters (Figure 4b,c). These results emphasized that networks in spring, plateau, or the source areas tended to be less complex and connected compared to those in autumn, plain, and the non-source areas, which underscored the impact of season, topography, and regional characteristics on prokaryotic taxa network structure. Additionally, both plain and non-source areas were characterized by a higher average betweenness centrality (Wilcoxon rank-sum tests, *p* < 0.01), suggesting that prokaryotic microbes in these regions occupied more central positions in the network [10] compared to those in plateau and source areas. This further demonstrated the structural differentiation of networks across various regions.

### 3.4. Potential Driving Factors of Bacteria–Archaea Co-Existence Patterns

The physicochemical properties of water samples varied significantly across seasons, landforms, and between the source and non-source areas (Figure S1). Water in autumn was characterized by significantly higher WT than spring (Spring: 11.14 ± 1.14 °C, Autumn: 21.71 ± 2.02 °C, one-way ANOVA, *p* < 0.001), while water in spring had greater concentrations of NH_4_^+^-N and metals like Ca, Fe, Al, and Ni (one-way ANOVA, *p* ≤ 0.001). Additionally, most environmental variables differed markedly among distinct terrains and locations. For example, both plateau and source areas had higher elevations and lower pH, TN, NO_3_^−^-N, and TP levels (one-way ANOVA, *p* < 0.05), while Ca was higher in these areas and Al was higher in both plain and non-source areas (one-way ANOVA, *p* < 0.01 for Ca; *p* < 0.05 for Al). NH_4_^+^-N, TN, and NO_3_^−^-N were much higher in the plain regions (one-way ANOVA, *p* < 0.001).

**Figure 5:**
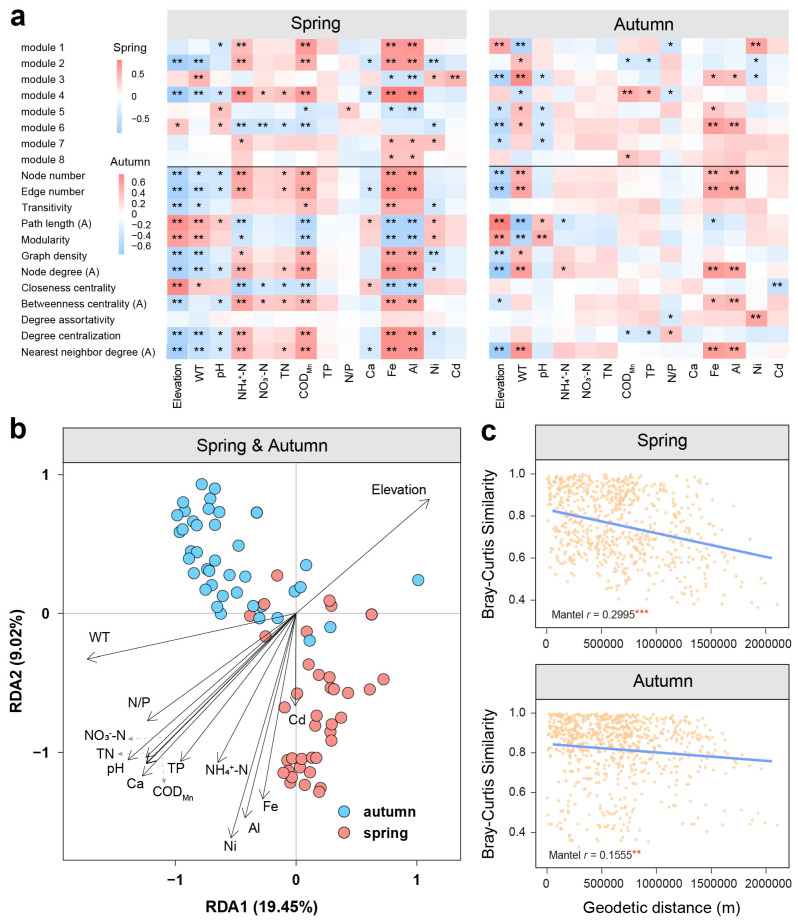
(**a**) Heatmap showing the Spearman correlations between environmental factors and total relative abundance of each module in the co-occurrence network and network topological properties. (**b**) Distance-based redundancy analysis (db-RDA) reveals the relative importance of environmental factors shaping bacterial and archaeal OTUs that occur in the network. (**c**) Geodetic distance-decay curves of Bray–Curtis similarities were calculated based on all the selected network topological parameters across all the sites in two seasons. Mantel Spearman’s *r* and *p* values are stated. All the asterisks denote the significance of correlations (*** *p* < 0.001, ** 0.001 < *p* < 0.01, and * 0.01 < *p* < 0.05).

A heatmap diagram was adopted to visualize Spearman’s correlations between environmental factors and network properties (i.e., modularity classes and topological features) for spring and autumn co-occurrence networks (Figure 5a). The results showed that the spring network may be more affected by environmental factors, with 47.9% of the major network modules and topological parameters significantly related to natural, nutrient, or metal factors, compared to 23.6% in autumn. Key factors potentially promoting the complexity and connectivity of the spring network contained NH_4_^+^-N, TN, COD_Mn_, and metals like Fe and Al, while WT, Fe, and Al had great impacts in autumn.

Distance-based redundancy analysis (db-RDA) was utilized to evaluate the relative impacts of physicochemical parameters and metalloids on the bacterial and archaeal communities that occurred in the co-existence network (Figure 5b). The first and second axes of db-RDA explained 19.45% and 9.02% variations, respectively. Specifically, WT (adj. *R*^2^ *=* 0.169, *p* = 0.001) and pH (adj. *R*^2^ *=* 0.135, *p* = 0.001) were identified as the primary seasonal factors influencing the members of the microbial networks. Other spatially-dependent factors including NH_4_^+^-N, NO_3_^−^-N, COD_Mn_, TP, and N/P also played significant but less influential roles on the OTUs in the network (adj. *R*^2^ *=* 0.062~0.115, *p* < 0.05). Metals like Al (adj. *R*^2^ *=* 0.078, *p* = 0.005) and Ni (adj. *R*^2^ *=* 0.082, *p* = 0.007) significantly affected the prokaryotic community structure in the network. These results were generally consistent with the results of Spearman correlation analysis in Figure 5a, which illustrated the critical roles of environmental factors (i.e., natural, nutrient, and metal factors) in shaping microbial interactions within the riverine ecosystems.

Distance-decay analysis revealed how geographic distance influenced the topological parameters of the spring and autumn networks (Figure 5c). The results showed that the geodetic distance better explained the network topology variations in spring (Mantel *r* = 0.2995, *p* < 0.001) rather than in autumn (Mantel *r* = 0.1555, *p* < 0.05). A larger slope for the distance-decay curve of the spring network was also observed. In the two-season network, significant Mantel correlations between geographic distance and most network topology parameters were found (*p* < 0.01) (Figure S2). In the spring network, geographic distance was significantly correlated with the variations of 10 parameters (Mantel *r* = 0.1021~0.2995, *p* < 0.05) (Figure S3). However, in the autumn network, only 5 out of 12 parameters were significantly correlated with the geographic distance (Mantel *r* = 0.1638~0.3674, *p* < 0.01) (Figure S4). This indicated the significant role of geographical distance in shaping the co-existence patterns of bacteria and archaea in spring along the Yangtze River, whereas the autumn network appeared to be less influenced by geographical distance, which was consistent with the results in Figure 5c.

## 4. Discussion

By conducting a comprehensive sampling in the continuous 6000 km aquatic body of the Yangtze River, we explored the co-existence patterns of planktonic bacteria and archaea across two seasons and distinct landforms in the world’s large river and identified the primary environmental factors affecting their co-existence. Previous studies mainly focused on co-existence network relationships between bacterial and archaeal communities in wetlands, soils, and sediments [14,15,34]. Our study filled the knowledge gap and demonstrated clear seasonal and spatial characteristics and driving mechanisms of bacterial and archaeal co-existence patterns in large natural rivers, which were influenced by physicochemical conditions and metal concentrations. This offered valuable insights into the ecological management of natural rivers.

By analyzing the microbial composition of modules and the O/R ratio between the major phyla in the co-occurrence networks, we revealed a preference for intradomain associations (bacteria–bacteria and archaea–archaea) over interdomain associations in spring, autumn, or both seasons (Figure 2a, Table 2). This suggested that microorganisms within the same taxonomic group might interact more frequently due to shared ecological niches and resource demands [35]. Season notably influenced the co-existence patterns of prokaryotic taxa, and archaea played a more crucial role than bacteria in the spring co-existence network as evidenced by their predominant presence of interphyla co-existence and archaeal OTUs in keystone species. Additionally, nine network topological parameters such as the number of nodes, the number of edges, and graph density were higher in autumn (Wilcoxon rank-sum tests, *p* < 0.05), which revealed a more complex and interconnected autumn network. This aligned with a study that indicated that increasing temperature could significantly enhance the network complexity of soil microbes [36]. This may be attributed to the lower WT and higher NH_4_^+^-N levels in spring (Figure S1). WT was a critical seasonal factor influencing the composition and diversity of planktonic bacterial and archaeal communities by affecting enzyme activity and inter-microbial interactions [37]. Lower WT might limit the bacterial diversity. For instance, bacterial communities in the Yangtze River water were significantly influenced by temperature, and more bacterial phyla preferred autumn with higher temperatures [9]. Whereas archaea, known for adapting to extreme conditions, could maintain high vitality under such conditions [3]. Moreover, as an important environmental factor of nutrients, NH_4_^+^-N may have a positive effect on the aggregation of archaeal communities, especially showing a positive correlation with certain phyla, like Bathyarchaeota, Aenigmarchaeota, YNPFFA, AK8, WSA2, and Lokiarchaeota [8,38,39]. A higher NH_4_^+^-N level in spring could lead to more linkages among archaeal phyla. River water in spring was characterized by elevated levels of Fe, Al, and Ni (Figure S1), potentially due to increased soil erosion from snowmelt and rainfall leading to higher discharge of these metals into the river [40]. Previous studies have implied that Fe/Al-bonded salts or minerals were easily formed as colloids and coagulants, as carriers for adsorbing microbes, nutrients, and macromolecular carbon [41]. This adsorptive effect might encourage the development of symbiotic microbial communities on Fe/Al-associated aggregates, especially for Bathyarchaeota adapting to Fe/Al-enriched environments [8]. Bathyarchaeota has been considered a “gatekeeper” to promote riverine archaeal diversity, stability, and predictability due to their metabolic versatility [8]. As a key element to form the methyl-coenzyme M reductase that catalyzes methane formation in all the methanogenic archaea, Ni has been confirmed to be positively correlated with the relative abundance of planktonic Euryarchaeota [8,42]. The increase in Ni concentration may impact the activity and abundance of methanogenic archaea, and further shift the microbial co-existence patterns.

Terrain and geographical location evidently affected the co-existence patterns of bacteria and archaea. By analyzing network topology parameters, we found that networks in plateau and source areas were much simpler, whereas networks in plain and non-source areas were more complex with microbes occupying more central positions in the network (Figure 4b,c). Moreover, network topological parameters of both seasons exhibited significant distance-decay patterns, suggesting a great spatial dependency of network associations, with a more significant pattern in spring (Figure 5c, Figures S2 and S3). This might be attributed to the typical determination effects of topography on environments like local sunlight, temperature, nutrient content, and human activity intensity, which are all commonly key factors affecting the composition of prokaryotic communities [43]. We found that the abundance of taxa in each module together with the network topology was more affected by environmental factors in spring than in autumn (Figure 5a), implying a critical role of the selection process on the co-existence, which can lead to a significant spatial turnover for the distance-decay pattern of prokaryotic taxa associations in spring. Various nutrient indices in spring (NH_4_^+^-N, TN, and COD_Mn_) and metal indices in both seasons were significantly and positively related to the network complexity. More carbon and nitrogen sources made microbial metabolism and activities more vigorous, which might increase the co-existence effects of prokaryotic taxa. Also, dispersal limitation might be more important to the distance-decay pattern with the lower flow in the spring dry season. Previous studies have demonstrated that bacterial communities in the Yangtze River water exhibited a significant distance-decay pattern, and landforms were the key factors shaping spatial variations of planktonic microbial community structures [8,9]. Similar results had been observed in other ecosystems [37,44,45].

## 5. Conclusions

This study advanced our understanding of the co-occurrence dynamics of planktonic bacteria and archaea over a 6000 km continuum of the Yangtze River and detailed the obvious seasonal and geographical variations of network patterns, offering a refined perspective of microbial community structure and function in natural river systems. We confirmed a pronounced preference for intradomain relations rather than interdomain relations for bacteria and archaea. Additionally, we found that the complexity of microbial networks varied with seasons: archaea were more prevalent and played more critical roles in spring, whereas autumn was characterized by more complex and connective co-existence patterns, reflecting the adaptive strategies of microbial communities to fluctuating environmental conditions. Moreover, the observed significant distance-decay patterns (more pronounced in spring) and the increased complexity and connectivity in plains and non-source areas imply the importance of considering spatial variability in ecological studies and conservation efforts. The critical roles of temperature, ammonium nitrogen, and specific metals such as Fe, Al, or Ni in influencing these co-existence patterns reveal the significant interplay between prokaryotic networks and physicochemical properties. Overall, these findings contribute to a broader understanding of the ecological co-existence network among prokaryotic taxa in aquatic environments and underscore the importance of investigating the mechanisms driving microbial interactions and their consequences for ecosystem health and stability.

## Figures and Tables

**Table 1:** Comparisons of topological properties of the Yangtze River prokaryotic microbial communities’ co-occurrence network with other types of networks ^a^.

Networks	Number of Nodes	Number of Edges (Spearman’s *r* > 0.6, FDR-*p* < 0.01)	MD ^b^	MDr ^b^	CC ^b^	CCr ^b^	APL ^b^	APLr ^b^	σ ^b^
Two seasons	838	16,309	0.658	0.010	0.642	0.047	3.763	2.111	7.663
Spring (dry season)	971	22,394	0.300	0.084	0.536	0.047	3.456	2.058	6.791
Autumn (wet season)	1071	10,192	0.688	0.160	0.457	0.018	3.892	2.689	17.541
Jiulong River microbial network	2194	44,680	0.672	0.087	0.511	0.0190	3.918	2.442	16.7628758
Jiulong River normal season subnetwork	1036	16,912	0.620	0.110	0.572	0.0320	4.540	2.314	9.110737885
Jiulong River dry season subnetwork	869	9078	0.423	0.151	0.526	0.0240	4.921	2.567	11.43265258
Jiulong River wet season subnetwork	1145	6879	0.549	0.205	0.415	0.0100	6.107	3.093	21.01842148
Marine microbial network	NA	NA	NA	NA	0.270	0.0440	2.990	2.620	5.37701429
Food webs	NA	NA	NA	NA	0.02–0.43	0.03–0.33	1.33–3.74	1.41–3.73	<1
Pollinator-plant networks	NA	NA	NA	NA	0.72–1.00	0.08–1.00	1.00–2.31	NA	NA
Microbial database network	NA	NA	NA	NA	0.501	NA	6.300	NA	NA
Functional microbial networks	NA	NA	NA	NA	0.10–0.22	0.028–0.099	3.09–4.21	3.00–3.84	<1
Caenorhabditis elegans, neural network	NA	NA	NA	NA	0.28	0.05	2.65	2.25	4.754716981
Escherichia coli, metabolic network	NA	NA	NA	NA	0.32–0.59	0.026–0.09	2.62–2.90	1.98–3.04	<1
Power grid	NA	NA	NA	NA	0.08	0.005	18.7	12.4	10.60962567
Actors	NA	NA	NA	NA	0.79	0.00027	3.65	2.99	2396.854389
Internet, domain level	NA	NA	NA	NA	0.18–0.3	0.001	3.70–3.76	6.18–6.36	>295

^a^: The topology of other co-occurrence networks was obtained from Hu et al. [28] and Steele et al. [33]. ^b^: Abbreviations: MD, modularity; CC, average clustering coefficient; APL, average shortest path length; σ, small-world coefficient σ = (CC/CCr)/(APL/APLr); the variables with r subscripts represent the properties of Erdös–Rényi random networks. NA: not available.

**Table 2:** Incidence of strong and significant intra- and inter-phylum co-occurrence patterns. The observed incidence (O) of co-occurrence between phyla was calculated as the number of observed edges divided by the total number of edges in the realistic networks, while the random incidence (R) was calculated by considering the node number of each phylum, and the random association among them. Only those observed co-occurrence patterns with O > 0.5% and O/R ratio > 1 are shown.

Groups	Phylum-1	Phylum-2	Phylum-1-Node Number	Phylum-2-Node Number	Edge Number	O(%)	R(%)	O/R-Ratio
Both seasons	Actinobacteria	Actinobacteria	94	94	732	4.49	1.25	3.60
	Bacteroidetes	Bacteroidetes	112	112	512	3.14	1.77	1.77
	Bathyarchaeota	Bathyarchaeota	68	68	1642	10.07	0.65	15.50
	Cyanobacteria	Cyanobacteria	43	43	112	0.69	0.26	2.67
	Euryarchaeota	Euryarchaeota	104	104	804	4.93	1.53	3.23
	Planctomycetes	Planctomycetes	29	29	94	0.58	0.12	4.98
	Proteobacteria	Proteobacteria	200	200	1190	7.30	5.67	1.29
	Thaumarchaeota	Thaumarchaeota	67	67	317	1.94	0.63	3.08
	Actinobacteria	Bacteroidetes	94	112	826	5.06	3.00	1.69
	Actinobacteria	Chloroflexi	94	13	155	0.95	0.35	2.73
	Actinobacteria	Cyanobacteria	94	43	213	1.31	1.15	1.13
	Actinobacteria	Gemmatimonadetes	94	5	95	0.58	0.13	4.35
	Actinobacteria	Planctomycetes	94	29	410	2.51	0.78	3.23
	Actinobacteria	Proteobacteria	94	200	1300	7.97	5.36	1.49
	Actinobacteria	Verrucomicrobia	94	12	123	0.75	0.32	2.34
	Bacteroidetes	Chloroflexi	112	13	104	0.64	0.42	1.54
	Bacteroidetes	Gemmatimonadetes	112	5	88	0.54	0.16	3.38
	Bacteroidetes	Planctomycetes	112	29	367	2.25	0.93	2.43
	Bacteroidetes	Proteobacteria	112	200	1131	6.93	6.39	1.09
	Bacteroidetes	Verrucomicrobia	112	12	132	0.81	0.38	2.11
	Bathyarchaeota	YNPFFA (archaea)	68	4	151	0.93	0.08	11.94
	Bathyarchaeota	Euryarchaeota	68	104	1544	9.47	2.02	4.69
	Bathyarchaeota	Thaumarchaeota	68	67	465	2.85	1.30	2.19
	Chloroflexi	Proteobacteria	13	200	166	1.02	0.74	1.37
	Cyanobacteria	Planctomycetes	43	29	105	0.64	0.36	1.81
	Gemmatimonadetes	Proteobacteria	5	200	152	0.93	0.29	3.27
	Planctomycetes	Proteobacteria	29	200	521	3.19	1.65	1.93
	Proteobacteria	Verrucomicrobia	200	12	165	1.01	0.68	1.48
Spring	Actinobacteria	Actinobacteria	74	74	218	0.97	0.57	1.70
	Bacteroidetes	Bacteroidetes	119	119	358	1.60	1.49	1.07
	Bathyarchaeota	Bathyarchaeota	115	115	4240	18.93	1.39	13.60
	Euryarchaeota	Euryarchaeota	137	137	1488	6.64	1.98	3.36
	Thaumarchaeota	Thaumarchaeota	87	87	391	1.75	0.79	2.20
	Aenigmarchaeota	Bathyarchaeota	8	115	453	2.02	0.20	10.35
	Aenigmarchaeota	Euryarchaeota	8	137	260	1.16	0.23	4.99
	Altiarchaeota	Bathyarchaeota	8	115	178	0.79	0.20	4.07
	Altiarchaeota	Euryarchaeota	8	137	126	0.56	0.23	2.42
	Bathyarchaeota	YNPFFA (archaea)	115	5	193	0.86	0.12	7.06
	Bathyarchaeota	Euryarchaeota	115	137	3772	16.84	3.35	5.03
	Bathyarchaeota	Hadesarchaea	115	2	144	0.64	0.05	13.17
	Bathyarchaeota	Lokiarchaeota	115	10	204	0.91	0.24	3.73
	Bathyarchaeota	Miscellaneous-Euryarchaeotal -Group (MEG)	115	5	127	0.57	0.12	4.64
	Bathyarchaeota	Thaumarchaeota	115	87	1814	8.10	2.12	3.81
	Bathyarchaeota	Woesearchaeota	115	23	146	0.65	0.56	1.16
	Euryarchaeota	Lokiarchaeota	137	10	177	0.79	0.29	2.72
	Euryarchaeota	Miscellaneous-Euryarchaeotal -Group (MEG)	137	5	113	0.50	0.15	3.47
	Euryarchaeota	Thaumarchaeota	137	87	956	4.27	2.53	1.69
	Euryarchaeota	Woesearchaeota	137	23	153	0.68	0.67	1.02
Autumn	Acidobacteria	Acidobacteria	33	33	70	0.69	0.09	7.45
	Actinobacteria	Actinobacteria	98	98	449	4.41	0.83	5.31
	Bacteroidetes	Bacteroidetes	141	141	281	2.76	1.72	1.60
	Bathyarchaeota	Bathyarchaeota	63	63	868	8.52	0.34	24.99
	Cyanobacteria	Cyanobacteria	67	67	334	3.28	0.39	8.49
	Euryarchaeota	Euryarchaeota	92	92	592	5.81	0.73	7.95
	Proteobacteria	Proteobacteria	295	295	925	9.08	7.57	1.20
	Thaumarchaeota	Thaumarchaeota	68	68	288	2.83	0.40	7.11
	Acidobacteria	Actinobacteria	33	98	68	0.67	0.56	1.18
	Acidobacteria	Planctomycetes	33	41	53	0.52	0.24	2.20
	Acidobacteria	Proteobacteria	33	295	324	3.18	1.70	1.87
	Actinobacteria	Bacteroidetes	98	141	381	3.74	2.41	1.55
	Actinobacteria	Chloroflexi	98	23	64	0.63	0.39	1.60
	Actinobacteria	Planctomycetes	98	41	77	0.76	0.70	1.08
	Bacteroidetes	Cyanobacteria	141	67	179	1.76	1.65	1.07
	Bacteroidetes	Planctomycetes	141	41	107	1.05	1.01	1.04
	Bathyarchaeota	YNPFFA (archaea)	63	4	57	0.56	0.04	12.72
	Bathyarchaeota	Cyanobacteria	63	67	209	2.05	0.74	2.78
	Bathyarchaeota	Euryarchaeota	63	92	606	5.95	1.01	5.88
	Bathyarchaeota	Thaumarchaeota	63	68	167	1.64	0.75	2.19
	Cyanobacteria	Planctomycetes	67	41	65	0.64	0.48	1.33
	Euryarchaeota	Thaumarchaeota	92	68	267	2.62	1.09	2.40
	Gemmatimonadetes	Proteobacteria	7	295	56	0.55	0.36	1.52
	Nitrospirae	Proteobacteria	5	295	66	0.65	0.26	2.52

## Data Availability

The raw data supporting the conclusions of this article will be made available by the authors on request.

## Supplementary Materials

The following supporting information can be downloaded at: https://www.mdpi.com/article/10.3390/microorganisms12071282/s1. Figure S1. Boxplots displaying the comparisons of environmental factors between spring and autumn (a), among plateau, mountain/hill, basin, and plain regions (b), and between the source and non-source regions (c); Figure S2. Geodetic distance-decay curves of Bray-Curtis similarities calculated based on the selected network topological parameters across all the sites in two seasons; Figure S3. Geodetic distance-decay curves of Bray-Curtis similarities calculated based on the selected network topological parameters across all the sites in spring; Figure S4. Geodetic distance-decay curves of Bray-Curtis similarities calculated based on the selected network topological parameters across all the sites in autumn; Table S1. Detailed information of the sampling sites along the Yangtze River.

## References

[1] Deng D., He G., Yang Z., Xiong X., Liu W. (2024). Activity and community structure of nitrifiers and denitrifiers in nitrogen-polluted rivers along a latitudinal gradient. Water Res..

[2] Gross A., Lin Y., Weber P.K., Pett-Ridge J., Silver W.L. (2020). The role of soil redox conditions in microbial phosphorus cycling in humid tropical forests. Ecology.

[3] Baker B.J., De Anda V., Seitz K.W., Dombrowski N., Santoro A.E., Lloyd K.G. (2020). Diversity, ecology and evolution of Archaea. Nat. Microbiol..

[4] van Wolferen M., Pulschen A.A., Baum B., Gribaldo S., Albers S.-V. (2022). The cell biology of archaea. Nat. Microbiol..

[5] Dang C., Wang J., He Y., Yang S., Chen Y., Liu T., Fu J., Chen Q., Ni J. (2022). Rare biosphere regulates the planktonic and sedimentary bacteria by disparate ecological processes in a large source water reservoir. Water Res..

[6] Ma L., Tan S., Liu H., Kao S.-J., Dai M., Yang J.-Y.T. (2021). Distribution and activity of Ammonia-oxidizers on the size-fractionated particles in the Pearl River estuary. Front. Mar. Sci..

[7] Yang J., Li G., Sheng Y., Zhang F. (2022). Response and contribution of bacterial and archaeal communities to eutrophication in urban river sediments. Environ. Pollut..

[8] Liu S., Lin Y., Liu T., Xu X., Wang J., Chen Q., Sun W., Dang C., Ni J. (2023). Planktonic/benthic Bathyarchaeota as a “gatekeeper” enhance archaeal nonrandom co-existence and deterministic assembling in the Yangtze River. Water Res..

[9] Liu T., Zhang A.N., Wang J., Liu S., Jiang X., Dang C., Ma T., Liu S., Chen Q., Xie S. (2018). Integrated biogeography of planktonic and sedimentary bacterial communities in the Yangtze River. Microbiome.

[10] Ma B., Wang H., Dsouza M., Lou J., He Y., Dai Z., Brookes P.C., Xu J., Gilbert J.A. (2016). Geographic patterns of co-occurrence network topological features for soil microbiota at continental scale in eastern China. ISME J..

[11] Liu J., Zhu S., Liu X., Yao P., Ge T., Zhang X.-H. (2020). Spatiotemporal dynamics of the archaeal community in coastal sediments: Assembly process and co-occurrence relationship. ISME J..

[12] Husnik F., Tashyreva D., Boscaro V., George E.E., Lukeš J., Keeling P.J. (2021). Bacterial and archaeal symbioses with protists. Curr. Biol..

[13] Wu Y., Zhou S., Li Y., Niu L., Wang L. (2024). Climate and local environment co-mediate the taxonomic and functional diversity of bacteria and archaea in the Qinghai-Tibet Plateau rivers. Sci. Total Environ..

[14] Chen B., Jiao S., Luo S., Ma B., Qi W., Cao C., Zhao Z., Du G., Ma X. (2021). High soil pH enhances the network interactions among bacterial and archaeal microbiota in alpine grasslands of the Tibetan Plateau. Environ. Microbiol..

[15] Yue Y., Tang Y., Cai L., Yang Z., Chen X., Ouyang Y., Dai J., Yang M. (2022). Co-occurrence relationship and stochastic processes affect sedimentary archaeal and bacterial community assembly in estuarine–coastal margins. Microorganisms.

[16] Liu S., Wang H., Chen L., Wang J., Zheng M., Liu S., Chen Q., Ni J. (2020). Comammox *Nitrospira* within the Yangtze River continuum: Community, biogeography, and ecological drivers. ISME J..

[17] Li L., Ni J., Chang F., Yue Y., Frolova N., Magritsky D., Borthwick A.G., Ciais P., Wang Y., Zheng C. (2020). Global trends in water and sediment fluxes of the world’s large rivers. Sci. Bull..

[18] Madsen E.L. (2011). Microorganisms and their roles in fundamental biogeochemical cycles. Curr. Opin. Biotechnol..

[19] Zheng T., Dang C., Zhong S., Sun W., Chen Q. (2021). Spatiotemporal distribution, risk assessment and source appointment of metal (loid) s in water and sediments of Danjiangkou Reservoir, China. Environ. Geochem. Health.

[20] Pires A.C., Cleary D.F., Almeida A., Cunha Â., Dealtry S., Mendonça-Hagler L.C., Smalla K., Gomes N.C. (2012). Denaturing gradient gel electrophoresis and barcoded pyrosequencing reveal unprecedented archaeal diversity in mangrove sediment and rhizosphere samples. Appl. Environ. Microbiol..

[21] Shan J., Ji R., Yu Y., Xie Z., Yan X. (2015). Biochar, activated carbon and carbon nanotubes have different effects on fate of 14C-catechol and microbial community in soil. Sci. Rep..

[22] Edgar R.C. (2013). UPARSE: Highly accurate OTU sequences from microbial amplicon reads. Nat. Methods.

[23] Edgar R.C., Haas B.J., Clemente J.C., Quince C., Knight R. (2011). UCHIME improves sensitivity and speed of chimera detection. Bioinformatics.

[24] Wang Q., Garrity G.M., Tiedje J.M., Cole J.R. (2007). Naive Bayesian classifier for rapid assignment of rRNA sequences into the new bacterial taxonomy. Appl. Environ. Microbiol..

[25] McDonald D., Price M.N., Goodrich J., Nawrocki E.P., DeSantis T.Z., Probst A., Andersen G.L., Knight R., Hugenholtz P. (2012). An improved Greengenes taxonomy with explicit ranks for ecological and evolutionary analyses of bacteria and archaea. ISME J..

[26] Quast C., Pruesse E., Yilmaz P., Gerken J., Schweer T., Yarza P., Peplies J., Glöckner F.O. (2012). The SILVA ribosomal RNA gene database project: Improved data processing and web-based tools. Nucleic Acids Res..

[27] Ju F., Xia Y., Guo F., Wang Z., Zhang T. (2014). Taxonomic relatedness shapes bacterial assembly in activated sludge of globally distributed wastewater treatment plants. Environ. Microbiol..

[28] Hu A., Ju F., Hou L., Li J., Yang X., Wang H., Mulla S.I., Sun Q., Bürgmann H., Yu C.-P. (2017). Strong impact of anthropogenic contamination on the co-occurrence patterns of a riverine microbial community. Environ. Microbiol..

[29] Liu S., Chen Q., Li J., Li Y., Zhong S., Hu J., Cai H., Sun W., Ni J. (2022). Different spatiotemporal dynamics, ecological drivers and assembly processes of bacterial, archaeal and fungal communities in brackish-saline groundwater. Water Res..

[30] Legendre P., Anderson M.J. (1999). Distance-based redundancy analysis: Testing multispecies responses in multifactorial ecological experiments. Ecol. Monogr..

[31] Dixon P. (2003). Vegan, a package of R functions for community ecology. J. Veg. Sci..

[32] Newman M.E. (2006). Modularity and community structure in networks. Proc. Natl. Acad. Sci. USA.

[33] Steele J.A., Countway P.D., Xia L., Vigil P.D., Beman J.M., Kim D.Y., Chow C.-E.T., Sachdeva R., Jones A.C., Schwalbach M.S. (2011). Marine bacterial, archaeal and protistan association networks reveal ecological linkages. ISME J..

[34] Wang B., Liu N., Yang M., Wang L., Liang X., Liu C.-Q. (2021). Co-occurrence of planktonic bacteria and archaea affects their biogeographic patterns in China’s coastal wetlands. Environ. Microbiome.

[35] Barberán A., Bates S.T., Casamayor E.O., Fierer N. (2012). Using network analysis to explore co-occurrence patterns in soil microbial communities. ISME J..

[36] Yuan M.M., Guo X., Wu L., Zhang Y., Xiao N., Ning D., Shi Z., Zhou X., Wu L., Yang Y. (2021). Climate warming enhances microbial network complexity and stability. Nat. Clim. Chang..

[37] Liu N., Wang B., Yang M., Li W., Shi X., Liu C.-Q. (2023). The different responses of planktonic bacteria and archaea to water temperature maintain the stability of their community diversity in dammed rivers. Ecol. Process..

[38] Zou D., Pan J., Liu Z., Zhang C., Liu H., Li M. (2020). The distribution of Bathyarchaeota in surface sediments of the Pearl River estuary along salinity gradient. Front. Microbiol..

[39] Zhou Z., Pan J., Wang F., Gu J.-D., Li M. (2018). Bathyarchaeota: Globally distributed metabolic generalists in anoxic environments. FEMS Microbiol. Rev..

[40] Xu X., Chen H., Hu J., Zheng T., Zhang R., Zhong H., Gao Q., Sun W., Chen Q., Ni J. (2022). Unveil the role of dissolved and sedimentary metal (loid) s on bacterial communities and metal resistance genes (MRGs) in an urban river of the Qinghai-Tibet Plateau. Water Res..

[41] Duan J., Gregory J. (2003). Coagulation by hydrolysing metal salts. Adv. Colloid Interface Sci..

[42] Thauer R.K., Kaster A.-K., Seedorf H., Buckel W., Hedderich R. (2008). Methanogenic archaea: Ecologically relevant differences in energy conservation. Nat. Rev. Microbiol..

[43] Wang J., Fu B., Qiu Y., Chen L. (2001). Soil nutrients in relation to land use and landscape position in the semi-arid small catchment on the loess plateau in China. J. Arid Environ..

[44] Liu L., Yang J., Yu Z., Wilkinson D.M. (2015). The biogeography of abundant and rare bacterioplankton in the lakes and reservoirs of China. ISME J..

[45] Logares R., Lindström E.S., Langenheder S., Logue J.B., Paterson H., Laybourn-Parry J., Rengefors K., Tranvik L., Bertilsson S. (2013). Biogeography of bacterial communities exposed to progressive long-term environmental change. ISME J..

