# Supplementary material for: Spatiotemporal Variations in Co-Occurrence Patterns of Planktonic Prokaryotic Microorganisms along the Yangtze River
Source: Microorganisms. 2024 Jun 24;12(7):1282. doi: 10.3390/microorganisms12071282 (PMC11278652; doi:10.3390/microorganisms12071282)
Supplement: Supplementary file 1 [file microorganisms-12-01282-s001.zip › microorganisms-2999072-supplementary.pdf]

# Supplementary Information for

# Spatiotemporal Variations in Co-occurrence Patterns of Planktonic Prokaryotic Microorganisms along the Yangtze River

**Authors:** Wenran Du[1,2], Jiacheng Li[2], Guohua Zhang[2], Ke Yu[1], Shufeng Liu[3]*

**Author affiliations:**

[1]School of Environment and Energy, Peking University Shenzhen Graduate School, Shenzhen 518055, China

[2]The Key Laboratory of Water and Sediment Sciences, Ministry of Education; Department of Environmental Engineering, Peking University, Beijing 100871, China

[3]College of Resources and Environmental Sciences, China Agricultural University, Beijing 100193, China

[*]**Correspondence:** Shufeng Liu, China Agricultural University, No. 2 Yuanmingyuan West Road, Beijing 100193, P.R. China. E-mail: liushufeng@cau.edu.cn

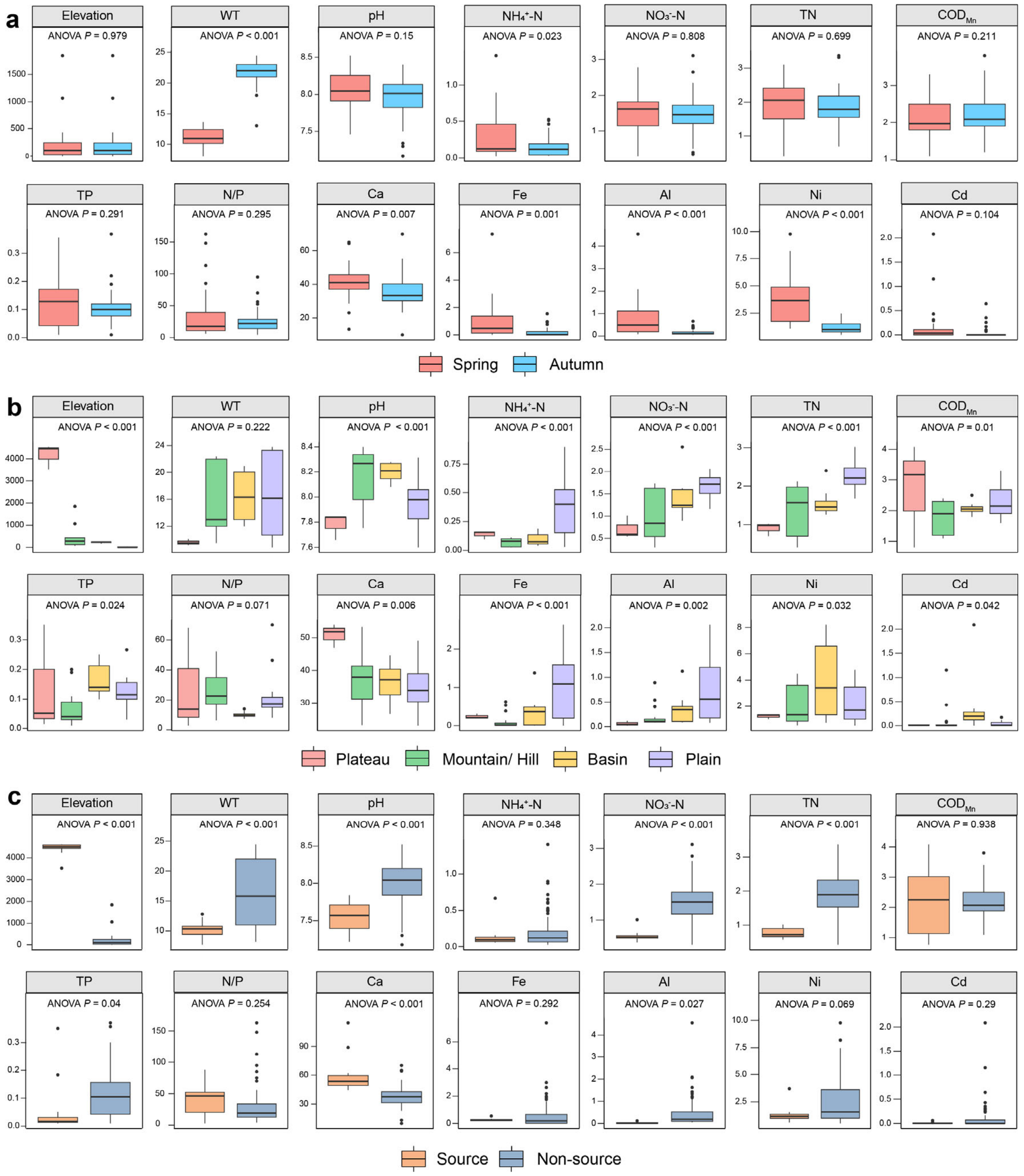


**Figure S1.** Boxplots displaying the comparisons of environmental factors between spring and autumn (**a**), among plateau, mountain/hill, basin, and plain regions (**b**), and between the source and non-source regions (**c**).

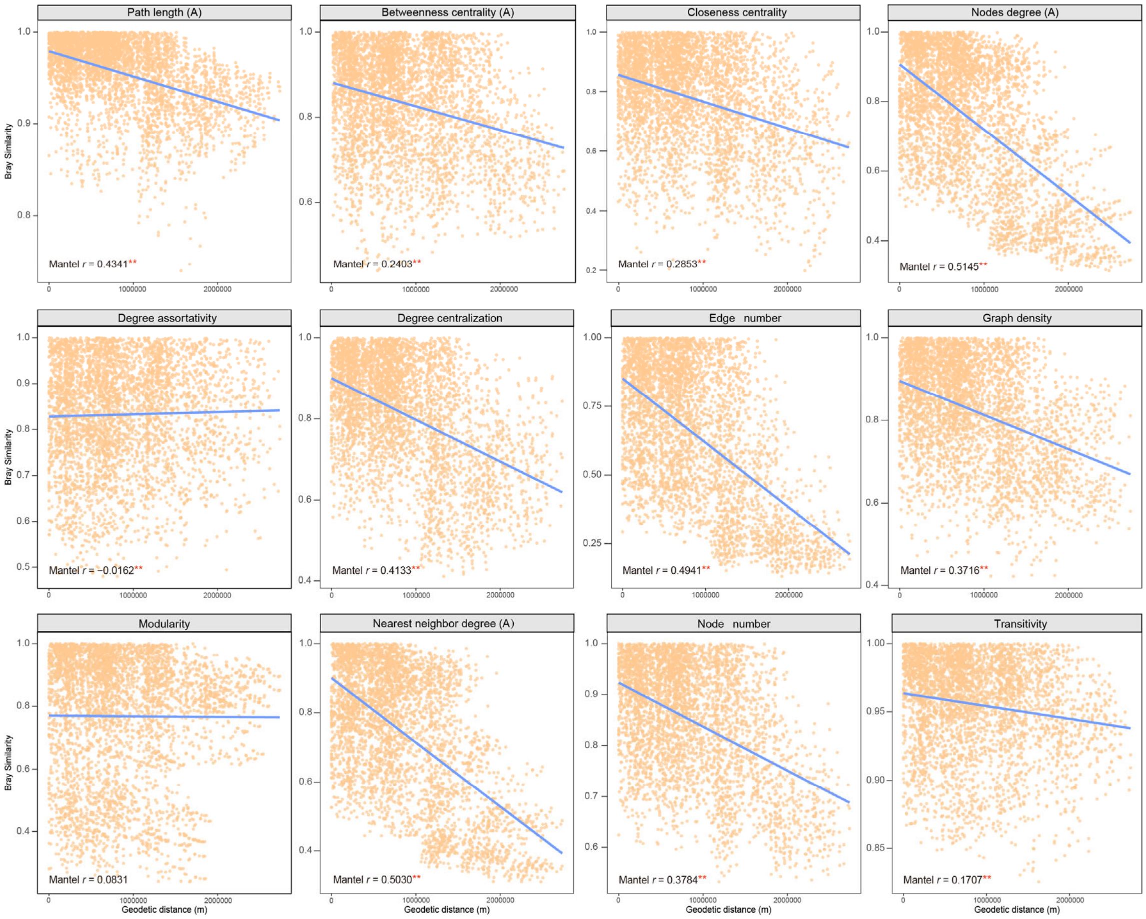


**Figure S2.** Geodetic distance-decay curves of Bray-Curtis similarities calculated based on the selected network topological parameters across all the sites in two seasons. Mantel Spearman's $r$ and $p$ values are stated. All the asterisks denote the significance of correlations (***$p < 0.001$, **$0.001 < p < 0.01$ and *$0.01 < p < 0.05$). The "A" in the brackets means "average".

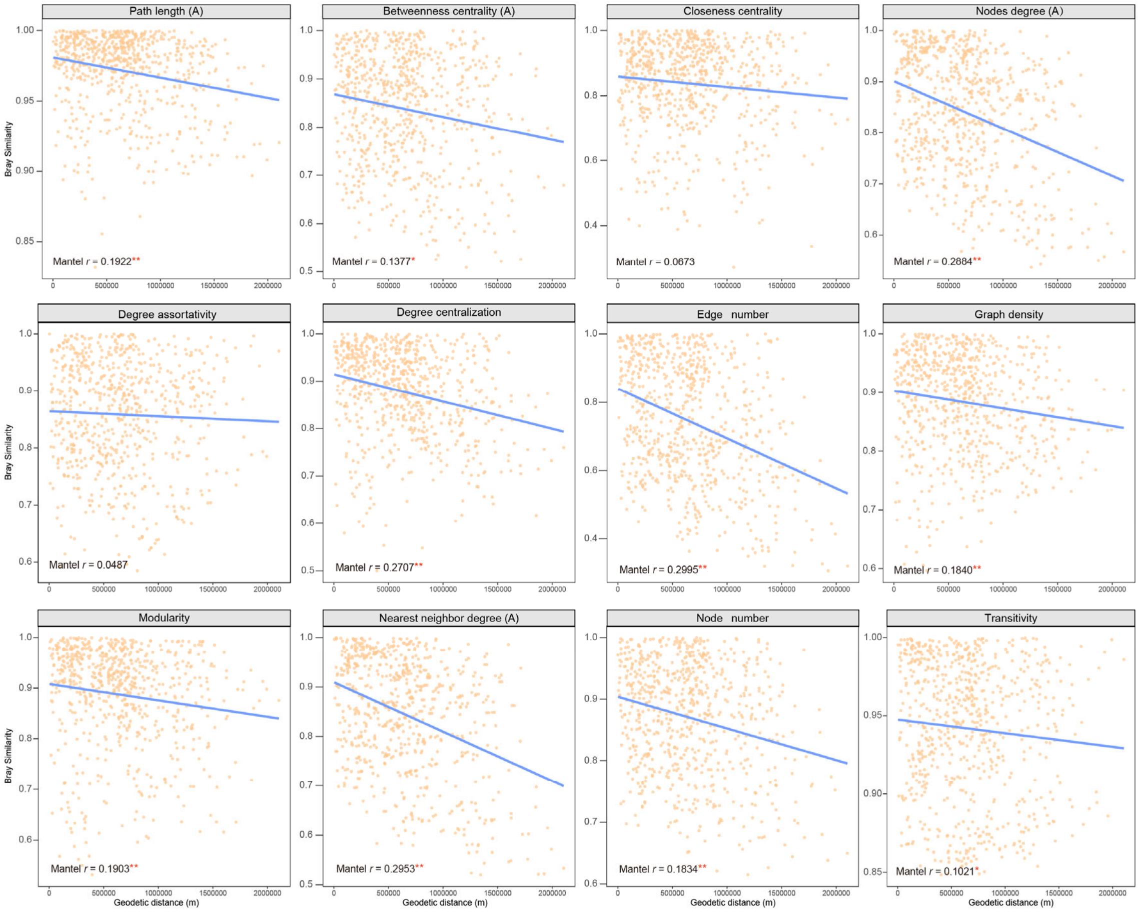


**Figure S3.** Geodetic distance-decay curves of Bray-Curtis similarities calculated based on the selected network topological parameters across all the sites in spring. Mantel Spearman's *r* and *p* values are stated. All the asterisks denote the significance of correlations (***$p < 0.001$, **$0.001 < p < 0.01$ and *$0.01 < p < 0.05$). The "A" in the brackets means "average".

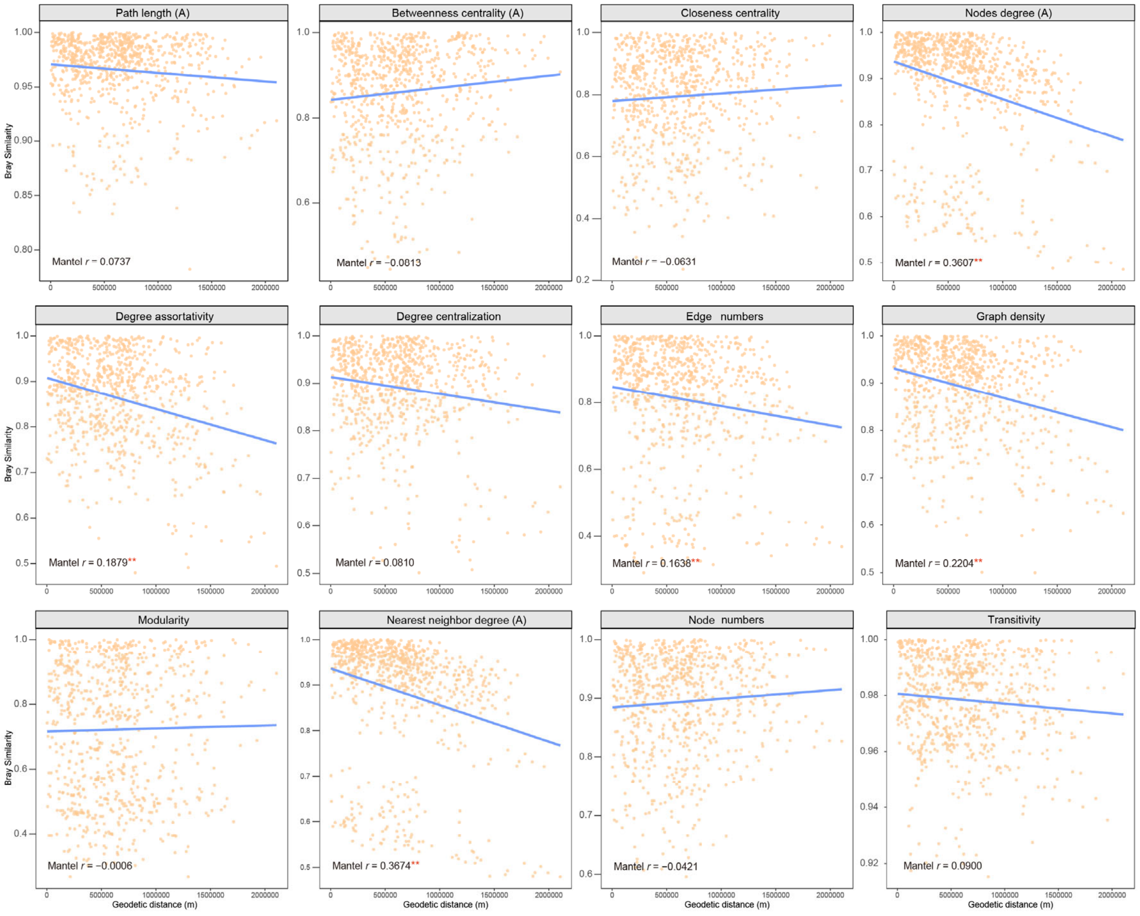


**Figure S4.** Geodetic distance-decay curves of Bray-Curtis similarities calculated based on the selected network topological parameters across all the sites in autumn. Mantel Spearman's *r* and *p* values are stated. All the asterisks denote the significance of correlations (***$p < 0.001$, **$0.001 < p < 0.01$ and *$0.01 < p < 0.05$). The "A" in the brackets means "average".

**Table S1.** Detailed information of the sampling sites along the Yangtze River.

| Site No. | Full name | Abbreviation | Longitude (°E) | Latitude (°N) | Mainstream/tributaries | Landforms |
|---|---|---|---|---|---|---|
| 1 | TuoTuoHe | TTH | 92.44 | 34.22 | Source-area mainstream | Plateau |
| 2 | TongTianHe | TOTH | 93.03 | 34.15 | Source-area mainstream | Plateau |
| 3 | ZhiMenDa | ZMD | 97.24 | 33.01 | Source-area mainstream | Plateau |
| 4 | ShiGu | SG | 99.98 | 26.88 | Mainstream | Mountain/Hill |
| 5 | PanZhiHua | PZH | 101.7 | 26.57 | Mainstream | Mountain/Hill |
| 6 | XiLuoDu | XLD | 103.66 | 28.25 | Mainstream | Mountain/Hill |
| 7 | PingShan | PS | 104.17 | 28.65 | Mainstream | Mountain/Hill |
| 8 | XiangJiaBa | XJB | 104.38 | 28.65 | Mainstream | Mountain/Hill |
| 9 | YiBin | YB | 104.65 | 28.77 | Mainstream | Basin |
| 10 | LuZhou | LZ | 105.55 | 28.9 | Mainstream | Basin |
| 11 | ZhuTuo | ZT | 105.85 | 29.02 | Mainstream | Basin |
| 12 | CunTan | CT | 106.6 | 29.62 | Mainstream | Basin |
| 13 | BaDong | BD | 110.4 | 31.04 | Mainstream | Mountain/Hill |
| 14 | MiaoHe | MH | 110.9 | 30.88 | Mainstream | Mountain/Hill |
| 15 | HuangLingMiao | HLM | 111.12 | 30.85 | Mainstream | Mountain/Hill |
| 16 | YiChang | YC | 111.28 | 30.69 | Mainstream | Mountain/Hill |
| 17 | ShaShi | SS | 112.26 | 30.29 | Mainstream | Plain |
| 18 | ChengLingJiLian | CLJL | 113.15 | 29.45 | Mainstream | Plain |
| 19 | LuoShan | LS | 113.32 | 29.67 | Mainstream | Plain |
| 20 | WuHan | WH | 114.32 | 30.62 | Mainstream | Plain |
| 21 | JiuJiang | JJ | 116 | 29.74 | Mainstream | Plain |
| 22 | DaTong | DT | 117.64 | 30.78 | Mainstream | Plain |
| 23 | WuHu | WHU | 118.34 | 31.46 | Mainstream | Plain |
| 24 | MaAnShan | MAS | 118.47 | 31.77 | Mainstream | Plain |
| 25 | NanJing | NJ | 118.94 | 32.17 | Mainstream | Plain |
| 26 | ZhenJiang | ZJ | 119.66 | 32.18 | Mainstream | Plain |
| 27 | XuLiuJing | XLJ | 120.96 | 31.77 | Mainstream | Plain |
| 28 | ChuMaErHe | CMEH | 94.47 | 35.08 | Source-area tributary | / |

| | | | | | | |
|---|---|---|---|---|---|---|
| 29 | DanQu | DQ | 93.83 | 32.88 | Source-area tributary | / |
| 30 | DeLieChuKa | DLCK | 91.76 | 33.67 | Source-area tributary | / |
| 31 | YanShiPing | YSP | 91.96 | 33.38 | Source-area tributary | / |
| 32 | DangQuDaQiao | DQDQ | 93.03 | 33.4 | Source-area tributary | / |
| 33 | MoQu | MQ | 93.69 | 34.14 | Source-area tributary | / |
| 34 | YaQu | YQ | 94.23 | 34.12 | Source-area tributary | / |
| 35 | KeQianQu | KQQ | 94.9 | 34.19 | Source-area tributary | / |
| 36 | LongBaoHe | LBH | 96.45 | 33.2 | Source-area tributary | / |
| 37 | GaoChang | GC | 104.41 | 28.8 | Tributary-Minjiang River | / |
| 38 | WuSheng | WS | 106.27 | 30.26 | Tributary-Wujiang River | / |
| 39 | BeiBei | BB | 106.39 | 29.88 | Tributary-Jialingjiang River | / |
| 40 | XiaoHeBa | XHB | 105.84 | 30.18 | Tributary-Jialingjiang River | / |
| 41 | LuoDuXi | LDX | 106.58 | 30.35 | Tributary-Jialingjiang River | / |
| 42 | WuLong | WL | 107.75 | 29.33 | Tributary-Jialingjiang River | / |
| 43 | XiaoXiTa | XXT | 111.3 | 30.78 | Tributary-Huangbohe River | / |
| 44 | YeMingZhu | YMZ | 111.3 | 30.75 | Tributary-Huangbohe River | / |
| 45 | ChengLingJi | CLJ | 113.13 | 29.42 | Tributary-Dongting Lake | / |
| 46 | NanZui | NZ | 112.31 | 29.06 | Tributary-Dongting Lake | / |
| 47 | ZhouWenMiao | ZWM | 112.06 | 28.92 | Tributary-Dongting Lake | / |
| 48 | XiangYin | XYN | 112.89 | 28.8 | Tributary-Dongting Lake | / |
| 49 | BaiHe | BH | 110.11 | 32.83 | Tributary-Hanjiang River | / |
| 50 | DanJiangKouBaXia | DJKBX | 111.52 | 32.51 | Tributary-Hanjiang River | / |
| 51 | XiangYang | XYA | 112.15 | 32.03 | Tributary-Hanjiang River | / |
| 52 | XianTao | XT | 113.45 | 30.58 | Tributary-Hanjiang River | / |
| 53 | JiJiaZui | JJZ | 114.23 | 30.57 | Tributary-Hanjiang River | / |
| 54 | TaoCha | TC | 111.65 | 32.66 | Tributary-Hanjiang River | / |
| 55 | HuKou | HK | 116.21 | 29.74 | Tributary-Poyang Lake | / |
| 56 | (P) LongKou | PLK | 116.21 | 29.02 | Tributary-Poyang Lake | / |
| 57 | (P) SheShan | PSS | 116.37 | 29.08 | Tributary-Poyang Lake | / |
| 58 | (P) HuKou | PHK | 116.22 | 29.75 | Tributary-Poyang Lake | / |
| 59 | (P) XingZi | PXZ | 116.04 | 29.44 | Tributary-Poyang Lake | / |
